# Supplementary material for: Association between oral microbiome and five types of respiratory infections: a two-sample Mendelian randomization study in east Asian population
Source: Front Microbiol. 2024 Apr 10;15:1392473. doi: 10.3389/fmicb.2024.1392473 (PMC11039966; doi:10.3389/fmicb.2024.1392473)
Supplement: Supplementary file 1 [file Data_Sheet_1.zip › Supplementary Materials/MR plots of saliva/Tonsillitis/s__Porphyromonas_endodontalis_mgs_3068/leave_one_out.pdf]

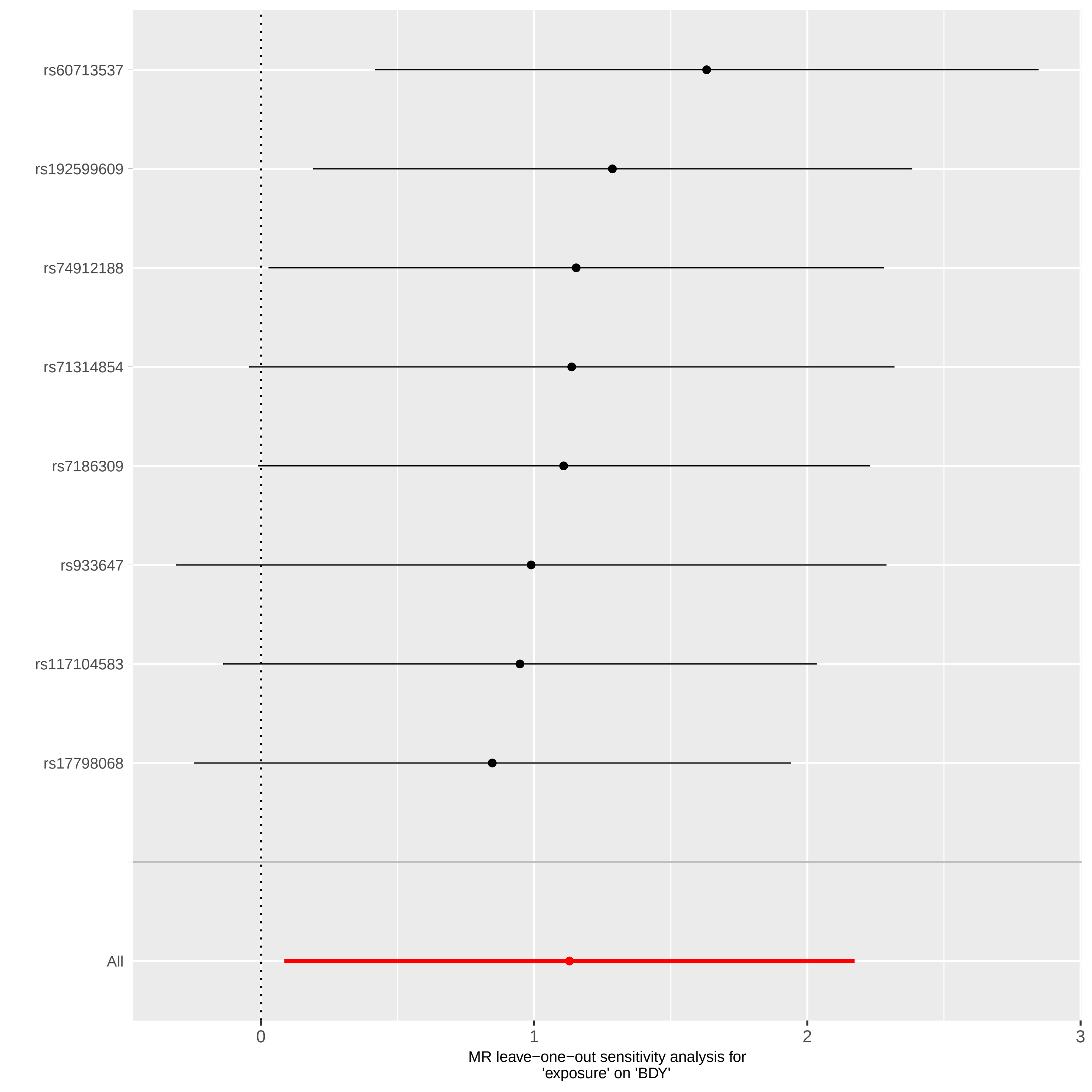
rs60713537
rs192599609
rs74912188
rs71314854
rs7186309
rs933647
rs117104583
rs17798068
All
0
1
2
3
MR leave−one−out sensitivity analysis for
'exposure' on 'BDY'
